# Heart rate variability in relation to cognition and behavior in neurodegenerative diseases: A systematic review and meta-analysis

**DOI:** 10.1016/j.arr.2021.101539

**Published:** 2022-01

**Authors:** Kathy Y. Liu, Thomas Elliott, Melanie Knowles, Robert Howard

**Affiliations:** a Division of Psychiatry, University College London, 149 Tottenham Court Road, London W1T 7NF, UK; b Camden & Islington NHS Foundation Trust, 4 St Pancras Way, London NW1 0PE, UK

**Keywords:** Heart rate variability, Neurodegenerative diseases, Dementia

## Abstract

Neurodegenerative diseases, which frequently present with neuropsychiatric symptoms related to prefrontal cortical dysfunction, can alter the integrity of the neural networks involved in central autonomic nervous system regulation, which is proposed to be indexed by heart rate variability (HRV). We systematically reviewed the characteristics, methodology and outcomes of 27 studies of HRV in relation to measures of cognition and behavior in neurodegenerative conditions, and assessed the strength of this relationship, cross-sectionally, across 18 studies. A significant, moderate effect was observed (r = 0.25), such that higher HRV was related to better cognitive and behavioral scores, which was not influenced by mean age or cognitive status. There was no evidence of small-study effects but we could not rule out publication bias, and other factors may have contributed to heterogeneity between studies. Our findings support the proposal that HRV may be a marker of self-regulatory processes in neurodegenerative conditions, and further research on this association is needed in relation to neuropsychiatric symptoms and alongside neuroimaging methods.

## Highlights

• Heart rate variability is proposed to be influenced by a neural network involved in autonomic regulation.
• Neurodegenerative processes alter the integrity of the central autonomic network.
• We reviewed studies of HRV and cognition/behavior in neurodegenerative diseases.
• We found a moderate correlation between HRV and measures of cognition/behavior.

## 1 Introduction

Heart rate variability (HRV), the beat-to-beat variation in heart rate, has long been considered a marker of cardiovascular risk (Electrophysiology Task Force of the European Society of Cardiology the North American Society of Pacing, 1996, Goldenberg Ilan et al., 2019), but is increasingly studied in relation to neural and cognitive processes. HRV can provide a measure of parasympathetic modulation (Berntson et al., 1997, Chapleau and Sabharwal, 2011, Levy, 1990) and studies have shown it is associated with a network of brain regions involved in autonomic nervous system regulation, known as the central autonomic network (Benarroch, 1993, Thayer et al., 2009). This network, which comprises prefrontal cortical (anterior cingulate, insula, orbitofrontal, and ventromedial cortices), limbic (central nucleus of the amygdala, hypothalamus), and brainstem regions, significantly overlaps with regions involved in ‘top-down’ self-regulation neural processes such as emotion regulation and executive functioning, leading to the proposal that vagally-mediated HRV may index these aspects of prefrontal cortical function (Porges, 2007, Thayer et al., 2009, Thayer and Lane, 2000).

In support of this, higher HRV has been linked to better cognitive function in healthy adults (Forte et al., 2019), including older individuals (mean age 60 years) (Frewen et al., 2013, Grässler et al., 2020, Schaich Christopher et al., 2020), and a recent meta-analysis found a small positive overall correlation (r = 0.09) between HRV indices and top-down self-regulation processes (including executive functioning, emotion regulation, and effortful or self-control) in mostly healthy participants across age groups (Holzman and Bridgett, 2017). A valid and easy-to-obtain physiological marker of emotion regulation capacity, such as HRV, would be useful to evaluate and monitor patients who have difficulty reliably self-reporting their emotional states, such as individuals with dementia, and may contribute to a greater understanding of the neurobiology underpinning neuropsychiatric symptoms.

Neurodegenerative disorders, including Alzheimer’s disease (AD), idiopathic Parkinson’s disease (PD), dementia with Lewy bodies (DLB) and frontotemporal dementia (FTD), can reduce the integrity of the central autonomic network and are associated with cardiovascular sympathovagal imbalance, abnormal emotional reactivity (Engelhardt and Laks, 2008, Femminella et al., 2014, Idiaquez and Roman, 2011, Seeley, 2010) and lowered HRV (Cheng et al., 2020). Impairments in executive dysfunction and emotion regulation have been hypothesized to underlie common and difficult-to-treat neuropsychiatric symptoms in dementia, including agitation, disinhibition and apathy (Chow et al., 2009, Lyketsos et al., 2004). Lower HRV measures reflect worse autonomic functioning in people with dementia versus controls (Cheng et al., 2020, da Silva et al., 2018), but the relationship between HRV and measures of cognition and behavior, as potential indicators of self-regulation capacity, in neurodegenerative conditions has not been previously reviewed. An understanding of the size and direction of any correlation between HRV and cognition/behavior in neurodegenerative disorders would clarify the potential of HRV as a biomarker for self-regulatory neural processes, and may contribute to the design of studies aiming to develop prevention and treatment strategies for neuropsychiatric symptoms in dementia.

The study aims to systematically review the characteristics, methodologies and findings of studies that reported any association between HRV and measures of cognition and/or behavior in patients diagnosed with a neurodegenerative disorder. A secondary aim was to estimate the overall effect size of the relationship, if sufficient and relevant data could be obtained.

## 2 Methods

### 2.1 Literature search

Online literature databases (Pubmed, PsychINFO, Embase and Web of Science) were searched up to 9th November 2020, which was updated on 26th March 2021, using the search terms “heart rate variability” AND (dementia OR Alzheimer disease OR Parkinson disease OR Lewy OR “mild cognitive impairment” or neurodegen*).

### 2.2 Inclusion/exclusion criteria

Studies were included if they were published, peer-reviewed articles in English on human subjects diagnosed with a neurodegenerative disorder, and reported findings on any association between HRV and measure(s) of cognition or behavior. Neurodegenerative disorders included diagnoses of AD, PD, DLB, vascular dementia, and multiple sclerosis. We also included studies of participants who were reported to have “dementia”, or diagnosed with preclinical AD or mild cognitive impairment (MCI) related to any of the neurodegenerative disorders defined above, including amnestic MCI (aMCI). Time and frequency-domain measures of HRV at rest, during task or HRV change/reactivity were included, and any related neuroimaging data was reported.

In our definition of neurodegenerative disorder, we did not include studies of traumatic brain injury, stroke, brain surgery, family history of late-onset AD, or cognitive impairment secondary to non-neurodegenerative conditions. We did not include studies of older adults who were reported to have subjective memory complaints but did not have a diagnosis of MCI. We excluded studies of physical or sensory functions (e.g. motor symptoms, physical fatigue and smell), autonomic impairment (e.g. cardiovascular performance or orthostatic intolerance), sleep architecture (e.g. using polysomnography), and studies solely focused on neuroimaging, disease severity, or functional status that did not report specific measures of cognition/behavior. For example, studies that only provided the United Huntington's Disease Rating Scale (UHDRS) total score were excluded, unless Part II (cognition) or Part III (behavior) subscales of the scale were reported. Studies that only reported the non-motor subscale (Part I) of the United Parkinson’s Disease Rating Scale (UPDRS), which includes cognitive, behavioral, physical and autonomic impairment symptoms, were excluded as this was judged not to be sufficiently specific to cognition/behavior. Case studies, conference abstracts, book chapters, letters, commentary, and dissertations were also excluded from the main analysis.

### 2.3 Data extraction

Two authors (out of TE, MK and KL) independently screened papers for inclusion based on their titles and abstracts, and subsequently extracted data on study characteristics, methods of HRV measurement and outcomes, related to any association between HRV and cognition/behavior, from relevant full-texts using a structured form. We were mainly interested in studies that analyzed a cross-sectional relationship between HRV indices and cognition/behavior. For included studies that described any or no correlation between HRV and cognition/behavior but did not provide quantitative data (i.e. a correlation coefficient), the corresponding author was emailed to request this information. Study characteristics were described using means and standard deviations (SD) or frequencies and proportions, as appropriate.

Studies that assessed any cross-sectional relationship between HRV and cognition/behavior, including intervention or pre-post studies, were assessed for quality using the NIH Quality Assessment tool for cross-sectional and observational studies (“NIH Study Quality Assessment Tools,” n.d.), which resulted in a quality rating for each study (low, high or some risk of bias). Discrepancies were resolved by discussion and/or re-extraction of the relevant data by KL.

### 2.4 Meta-analysis

Studies that provided a cross-sectional correlation effect size (Pearson’s r or beta-coefficient) between vagally-mediated HRV (Shaffer and Ginsberg, 2017) (defined below) and any cognition/behavior measures were included in a random effects meta-analysis model to compute a weighted effect size and confidence interval across studies using the ‘dmetar’ package (version 0.0.9000) (Harrer et al., 2019) in R version 3.6.3. For some studies that reported a standardized beta coefficient between + /− 0.5, these were converted to Pearson’s r using established formulae (Peterson and Brown, 2005), and studies that reported beta-coefficients outside this range were not included in the meta-analysis. Effect sizes that were not reported or provided as a result of requests for information from significant studies were not included in the meta-analysis, and for non-significant studies, effect size estimates were imputed with a value of r = 0 as a conservative estimate. Effect sizes were scaled so that higher scores indicated better cognitive or behavioral performance. Pearson’s r correlation values of 0.10 were defined as small effects, 0.20 as typical, and 0.30 as large (Gignac and Szodorai, 2016) and results were evaluated at the p = 0.05 level.

Although HRV is under predominant vagal control, certain HRV measures, described to be vagally-mediated, are interpreted to be specific to parasympathetic function. For example, the high-frequency (HF) component to HRV is believed to index the vagal modulation of heart rate (Electrophysiology Task Force of the European Society of Cardiology the North American Society of Pacing, 1996, Shaffer and Ginsberg, 2017). The HF variations in heart rate correlate highly with the root mean square of successive R-R interval differences (RMSSD) and percentage of successive N-N intervals that differ by more than 50 ms (pNN50), and RMSSD is preferred to pNN50 because it has better statistical properties (Electrophysiology Task Force of the European Society of Cardiology the North American Society of Pacing, 1996). Measures that reflect respiratory sinus arrhythmia (RSA), the respiratory-driven changes in heart rate via the vagus nerve, also provide a measure of vagally-mediated HRV, and include the average difference between the highest and lowest heart rates during each respiratory cycle (HRmax-HRmin), the R-R interval variation (RRIV) (Persson and Solders, 1983) and the ratio of the longest R-R interval during expiration to the shortest R-R interval during inspiration (expiration/inspiration or E/I ratio) (Chaswal et al., 2018).

Only one effect size estimate for each study contributed to the pooled effect size, thus if a study reported multiple relevant effect sizes, vagally-mediated HRV measures were preferentially chosen for inclusion in the meta-analysis in the following order: resting HF, RMSSD, pNN50, RSA-related measures including HRmax-HRmin, RRIV, and E/I; followed by the same indices for task-related HRV and HRV reactivity or change. We did not include the standard deviation of N-N (SDNN) or R-R (SDRR) intervals, low frequency (LF), ultra-low-frequency (ULF), very-low-frequency (VLF) band power or LF/HF ratio in our analyses of vagally-mediated HRV, as both parasympathetic and sympathetic nervous system activity have been proposed to contribute to these indices (Shaffer and Ginsberg, 2017). For cognition/behavior, measures of executive function (or an average value if multiple executive functions were reported) were preferentially selected, followed by global cognition and any other cognitive or behavioral measures.

Study heterogeneity was measured using the I^2^ statistic. Forest and funnel plots were created to graphically represent effect sizes and visualize small-study effects respectively. Outlier effect sizes that differed significantly from the pooled effect (i.e. the lower bound of the 95% confidence interval of the study effect size was higher than the upper bound of the pooled effect 95% confidence interval, and/or the upper bound of the 95% confidence interval of the study effect size was lower than the lower bound of the pooled effect 95% confidence interval) were detected using the ‘dmetar’ statistical package to explore the effect of their exclusion on the pooled effect size and study heterogeneity.

### 2.5 Post-hoc analyses

Post-hoc analyses included a meta-regression to explore whether the mean age or Mini-Mental State Examination (MMSE) score of study participants were associated with effect size differences. Estimates of MMSE were imputed for the studies that only reported the Montreal Cognitive Assessment (MoCA) (Bergeron et al., 2017) or Clinical Dementia Rating-Sum of Boxes (CDR-SB) (Balsis et al., 2015) scores. We also conducted separate meta-analyses to obtain specific pooled effect size estimates for resting vagally-mediated HRV, vagally-mediated HRV reactivity, and executive function. Although it has been recommended that spontaneous breathing in any posture (e.g. sitting, supine or standing) held for at least 5 min can provide a baseline HRV measurement (Laborde et al., 2017), we conservatively defined resting HRV as HRV measured during spontaneous breathing in a sitting or supine position. Studies that reported resting HRV without describing participants’ posture during measurement, or HRV measured during standing, orthostatic challenge, deep breathing or valsalva maneuver were excluded from the post-hoc meta-analysis of resting HRV.

One author (KL) screened conference abstracts and dissertations that were unlinked to published articles from the literature search results, and extracted any reported correlations and effect sizes, to explore the potential presence of publication bias.

## 3 Results

### 3.1 Identification and characteristics of included studies

**Fig. 1:**
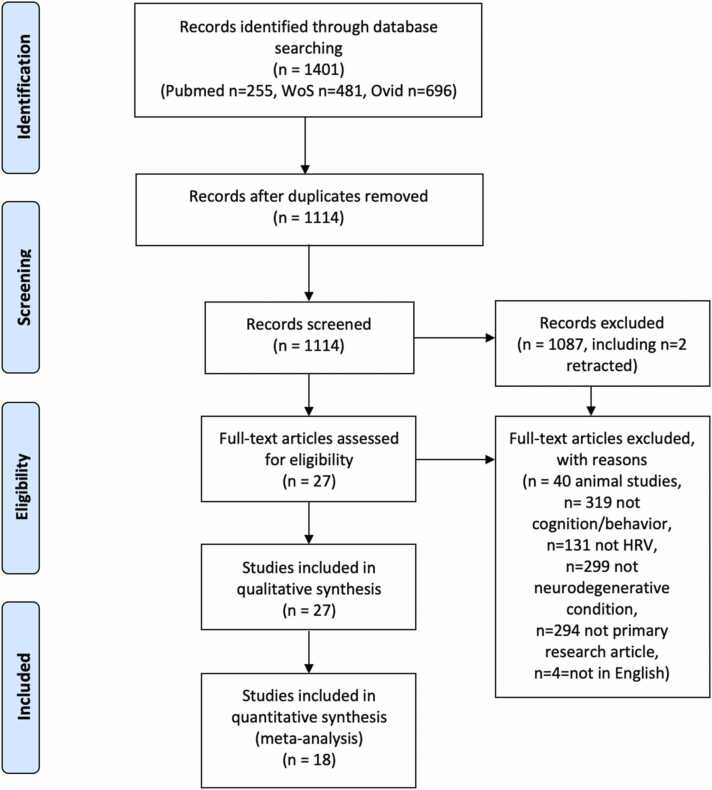
PRISMA flow diagram.

Literature searches identified 1114 potential studies, of which 27 met inclusion criteria for data extraction (Fig. 1, PRISMA flow diagram). The characteristics, HRV measurements and outcomes of included studies are displayed in Table 1. The details of any neuroimaging findings reported by studies are displayed in Supplemental Table 1.

**Table 1:** Characteristics and findings of included studies. Sample size, age, and sex data are reported only in relation to the analyzed group whose correlation statistic is provided. Correlation statistics are for HRV at rest, unless specified otherwise. Correlation values used in the meta-analysis are shown in bold, and these were sometimes an average of reported measures (*avg*). For studies that reported a neuroimaging measure(s) (Y), details of the neuroimaging findings are reported in Supplemental Table 1.

Study	Population	Diagnostic criteria	Study design	N	Exclusions (comorbidities/medications)	Mean age (years)	% female	Baseline cognition	Source of RR intervals	Conditions	Recording length	HRV measure (s)	Cognitive/behavioral measure (s)	Correlation statistic (cognitive/behavior)	Neuroimaging measure (s)	Covariates/confounders reported to be included in analysis
*Studies included in meta-analysis*																
(de Vilhena Toledo and Junqueira, 2010)	AD	Y	CS	22	N	79.6	90.9	MMSE 6.8	ECG	Resting supine and standing	Short-term; 5 min	SDNN, CV; RMSSD, ULF, VLF, LF, HF, LF/HF, TP, absolute power	MMSE and CAMCOG	[Supine] MMSE and SDNN r = −0.03, CV r = −0.10, pNN50 r = 0.31, **RMSSD r = 0.28**; [Supine] CAMCOG and SDNN r = 0.009, CV r = −0.02, pNN50 r = 0.39, RMSSD r = 0.33; [Standing] MMSE and SDNN r = 0.31, CV r = 0.2, pNN50 r = 0.31, RMSSD r = 0.38, [standing] CAMCOG and SDNN r = 0.37, CV r = 0.27, pNN50 r = 0.43, RMSSD r = 0.47.	N	N
(Zulli et al., 2005)	aMCI, AD, HC	Y	CS	101	AchEIs, HF, coronary artery or valvular disease, DM; MDD or CVD; TBI; B12, folate or thyroid abnormalities.	70.6	61.3	MMSE 25.1	ECG	24 hr ambulatory (minimum 18 hrs), 10 am-10 pm, supine between 3 and 4 pm	Long term; 24 hr	RMSSD, LF, HF, LF/HF, SDRR, SDARR; SDRR idx; mean 5 min values	MMSE; ADAS-Cog	In multivariate models, MMSE and HF β = 0.41 and 0.38 (***avg β = 0.4**)* (p < 0.01), ADAS-Cog and HF-HRV β = −0.24 and − 0.21(p > 0.05), ADAS-cog and LF/HF β = 0.17 (p = 0.19).	N	N
(Quinci, M.A., Astell, A.J. 2021)	Dementia, including AD, VD and mixed	N	CS	11	N	90	70.0	MoCA 8.2	Wrist-worn monitor; non-dominant hand	Upright sitting position, eyes open during	Short-term; two approx. 2 min recordings	RMSSD; lnRMSSD	HADS-A score	NR but raw data for each participant was reported and correlation could be calculated. **r = −0.319,** p = 0.369	N	N
(Nonogaki et al., 2017)	AD	Y	CS	78	Beta-blockers, medical causes of dementia, cardiac arrhythmias, cardiac surgery, ablation or implantation of a pacemaker	77.1	64.1	MMSE 22.4	ECG	Supine	Short-term; 5 min	LF, HF, LF/HF	MMSE and cognition composites (total z-scores of immediate and delayed word recall, logical memory I and II from Wechsler Memory Scale-Revised, category fluency, letter fluency, digit symbol subtest of Wechsler Adult Intelligence Scale revised, Clock drawing test and Stroop Colored-Word test)	MMSE and LF/HF: β = −0.31; LF: β = −0.1; HF: β = 0.18. Cognition composite and LF: β = −0.01; HF: β = 0.22; LF/HF: β = −0.26. Composite memory and LF: β = −0.02; HF: β = 0.25; LF/HF: β = −0.03. Composite executive function+processing speed and LF: β = 0.02, **HF: β = 0.07**; LF/HF: β = −0.06.	N	Y - age, sex, education years, HTN, DM, AChEIs
(Guo et al., 2016)	bvFTD	Y	CS	17	Antihypertensives with known effects on autonomic system e.g. “beta-blockers, calcium channel blockers, anticholinergics”.	59.1	29.4	CDR-SB 7.7	Pulse oximetry	During rest, supine (task-free fMRI scanning)	Short term; 16 min	Natural log of the variance in the IBI time series (lnHRV); an estimate of sympathetic-related variability [Toichi cardiac sympathetic index (CSI)], an estimate of parasympathetic-related variability [Toichi cardiac vagal index (CVI)], and RSA [natural log of the HF variance of IBI time series)	NEO-PI-3 subscales inc. agreeableness and positive emotion (the tendency to experience positive emotions such as happiness), NPI subscales apathy and disinhibition.	Agreeableness was related to cardiac vagal tone, CVI r = 0.54 (p = 0.019), or lnHRV r = 0.42 (p = 0.023). Cardiac vagal tone was not sig. ass. with positive emotion, r = 0.15, (p = 0.2). No correlation with NPI apathy or disinhibition r < 0.3, (P > 0.3), ***avg r*****=*****0.35.***	Y	Y - age, gender, BMI
(Sharma et al., 1999)	HD	Y	CS	22	N	49	59	9 had dementia	EMG	During rest and 15 s valsalva	Short term; NR	RRIV (=b/a×l00 (b=RR range i.e. difference between shortest and longest RR interval, a=average of longest and shortest RR interval)	UHDRS subscales	Resting HRV and verbal fluency r = 0.2, p > 0.5; symbol digit r = 0.6, p < 0.1; Stroop total r = 0.4, p > 0.5; ***avg exec r = 0.4**;* total behaviour score r = −0.3 (p < 0.5). Valsalva HRV and verbal fluency r = 0.5, p < 0.5; symbol digit r = 0.6, p < 0.1; Stroop total r = 0.5, p < 0.5*;* total behaviour score r = −0.4	N	N
(Del Pino et al., 2020)	LBD, PD	Y	CS	61	DM, heart diseases potentially influencing hemodynamic measures, or any other neurological disorder	61.5	38.7	NR	ECG	Resting supine	Short-term; 10 min	The expiratory-to-inspiratory ratio (E/I) during DBT, and the Valsalva ratio (longest R–R interval in phase IV divided by shortest R–R interval between phase II and the very beginning of phase III).	Various cognitive functions	Valsalva ratio and general cognition r = 0.31, perception r = 0.36, executive functions r = 0.3, attention r = 0.35, verbal fluency r = 0.31, verbal memory r = 0.3, visual memory r = 0.35, visuospatial r = 0.45. E/I ratio and general cognition r = 0.28, perception r = 0.48, exec functions r = 0.5, attention r = 0.49, verbal fluency r = 0.42, verbal memory r = 0.44, visual memory r = 0.57, apathy r = −0.4, depression r = −0.26, ***avg exec r = 0.4***	N	N
(Lin et al., 2016)	aMCI	N	CS	19	No major cardiovascular conditions	72.3	42	MoCA 24.5	ECG	Rest and cognitive stress task, between 9 and 11 am, abstained from exercise, caffeine, medications.	Short-term; 10 mins during rest and 60 min during cognitive task	HF, modelled over last minute baseline and 60 min tasks with a quadratic model to calculate parameter coefficients for vertex (time when HRV starts rebounding), change over the tasks, bottom and initial level.	Mental fatigability (MF)	MF was significantly related to vertex, r = 0.44 (p = 0.03) and change, **r = −0.41** (p = 0.04), but not the bottom or initial level of the HF-HRV quadratic model. MF was not significantly correlated with average HF-HRV over 60 min cognitive stress task, **r = −0.15** (p = 0.27).	Y	Y - medications, medical conditions, disease severity
(Lin et al. 2017)	aMCI, HC	Y	CS	38	Not stabilised on anti dementia medications, severe cardiovascular or inflammatory diseases inflammatory, uncontrollable psychiatric disorders, uncontrollable HTN, or MRI contraindications	72.6	58.3	MoCA 25.2	ECG	At rest, during task and recovery	Short-term; 10–13 mins	HF; also reactivity (HF-HRV during task minus HF‐HRV at rest)	Executive function (Stroop Color Word and Dual 1–back task,) and episodic memory	Episodic memory and resting HF-HRV r = −0.46; reactivity: r = 0.48. Executive function and resting HF-HRV: **r = −0.34**; reactivity: **r = 0.29**	Y	Y - group (clinical phenotype)
(Kim et al., 2018)	MCI (MCI-AD and MCI-DLB)	N	CS	55	Beta-blockers, thyroxine, focal brain lesions, multiple lacunar infarctions or diffuse white matter hyperintensity, clinical diagnosis of PD, DM or cardiac diseases	70.3	54.3	MMSE 25	ECG	Supine between 8 am and 12 pm. Avoided caffeine > 10hrs and nicotine > 1 hr	Short term; NR	SDNN, RMSSD, LF, HF, TP	Various cognitive functions. Fronto-executive function was tested using phonemic generative naming (PGN), COWAT for animals/supermarket, Stroop word and Stroop color tests.	PGN and SDNN r = 0.150; RMSSD r = 0.183; TP r = 0.067; LF r = −0.057; HF r = 0.091; LF/HF r = −0.167. COWAT animal and SDNN r = 0.204; RMSSD r = 0.145; TP r = 0.197; LF r = 0.207; HF r = 0.232; LF/HF r = −0.071. COWAT supermarket and SDNN r = 0.389; RMSSD r = 0.348; TP r = 0.254, LF r = 0.177, HF r = 0.179, LF/HF r = 0.009. Stroop word and SDNN r = −0.036; RMSSD r = −0.026, TP r = −0.018; LF r = −0.018, HF r = 0.033, LF/HF r = 0.068. Stroop color and SDNN r = 0.208; RMSSD r = 0.283; TP r = 0.229, LF r = 0.229, HF r = 0.29, LF/HF r = −0.218, ***RMSSD/HF avg exec r = 0.176***	Y	Y - age, gender, education
(Nicolini et al., 2020)	MCI (amnestic and non-amnestic)	Y	CS	82, 93	Beta-blockers, alpha blockers, centrally-acting calcium-channel blockers, class I and III antiarrhythmic drugs, digoxin, TCAs, SSNRIs, atypical antidepressants, antipsychotics and AChEIs.	79.5, 78.9	68.3, 67.7	MMSE 25.8, 27.5	ECG	Supine resting and standing, between 8.30 and 11 am, abstained from caffeine, alcohol, nicotine, vigorous exercise < 12hrs	Short term; 15 min supine resting, 5 min active standing taken after 10 min standing	Change in (Δ) nLF, HF and LF/HF (active standing measure minus baseline measure)	Various cognitive tests	For aMCI, ΔLFn and ΔLF/HF exhibited a significant positive correlation with the prose-delayed recall Z-score. No significant correlations were found for standing HF (r = 0.052). For naMCI ΔLFn and ΔLF/HF exhibited a significant negative correlation with the Digit Cancellation test (DCT) and executive functioning Z-scores. No significant correlations were found for standing HF. For standing HF and exec functions r = 0.161.	Y	Y
(Lin et al., 2020)	aMCI	Y	Controlled intervention	84	Anti-dementia drugs unless stable doses > 3 months; change in antipsychotic, antiseizure, antidepressant or anxiolytic medications in the past 3 months; MDD; MRI contraindications; major vascular disease e.g., stroke, myocardial infarction, CHF.	74.7	48.3	MoCA 24.1	ECG.	Baseline during rest and task, post-test (1 week after intervention) and 6 months post test	Short term; NR	HF	Useful Field of View (UFOV) - a measure for processing speed and attention, and working memory	Across two MCI groups (one of which received a processing speed/attention targeted intervention) from baseline to post-test, improvement (i.e., decrease in reaction time) in UFOV over time was related to improvement in HF-HRV_task when controlling for HRV_rest. (B=−0.33, p = 0.0577) These results did not change when controlling for age, sex, and a AD-related cortical thickness score (**B = −0.31, p = 0.068**).	Y	Y - age, sex, cortical thickness
(Lin et al. 2017)	aMCI	Y	CS	21	Antidepressants or anxiolytics, AChEIs/memantine started < 3months	73	53	MoCA 25	ECG	During cognitive training or mental leisure activities	Short term; 60 min	HF response to training over 60 mins, quadratic model ( rate of change, minimum and initial level).	Useful Field of View (UFOV) - a measure for processing speed and attention, and working memory	HF-HRV quadratic term (rate of change) correlated with changes in UFOV, r = 0.39, and working memory, r = 0.33, ***cog avg= 0.36***. Combined two treatment groups who received cognitive training or mental leisure activities.	Y	N
(McDermott et al., 2019)	aMCI, HC	Y	CS and case-control	17, 22	N	73.9, 71.2	52.9, 63.6	MoCA 24.1, 26	ECG	Rest, during cognitive stress task and recovery	Short-term; 10 min rest, 20 min during cognitive stress (Stroop and Dual 1-back) task, 10 min recovery	HF derived over 20 s intervals, natural log transformed; quadratic term from quadratic model extracted.	Perceived Stress Scale (PSS), a measure of chronic stress	Combining HC and aMCI groups together, there was a correlation between PSS and HF-quadratic (r = .32, p = 0.045). This correlation also held up separately for HC (r = 0.46, p = 0.03), but not aMCI (**r = 0.17**, p = 0.51).	Y	N
(Sander et al., 2019)	MS	Y	CS	53	Corticosteroid use, pregnancy, non-MS related psychiatric disease, relapse < 4wks	50.1	79.2	NR	Pulse oximetry	During acoustic vigilance task	Short-term; 20 min	SDNN, RMSSD, pNN50, VLF, LF, HF	Trait and time-on-task fatigue	Only significant results shown/reported. Trait fatigue and VLF: β = −0.573; and **HF: β = 0.404** Time-on-task fatigue and pNN50: β = 0.994, and SDNN: β = −0.793.	N	N
(Combs et al., 2018)	PD	N	CS	31 (29 analysed)	Cardiac pacemaker	66.7	55	FSIQ 100.7	ECG	Resting sitting	Short term; 8 min	RMSSD	Various cognitive functions. Executive function measured by TMT-B, COWAT, Animal category test, IGT.	RMSSD and TMT-B r = 0.49; COWAT r = −0.07; animals r = −0.01; IGT r = −0.12, ***avg exec r = 0.0725***	N	N
(Bidikar et al., 2014)	PD	Y	CS	30	Cardiovascular disease and abnormal ECG	NR (range 55–70)	NR	Disease duration 7 yrs	ECG	Resting supine between 9 and 11 am during “on” phase. Avoided caffeine and nicotine > 6hrs	Short-term; 30 s	HRmax-HRmin (mean over six breathing cycles)	NMSS scale (9 domains including attention/memory, perceptual problems/hallucinations, mood/cognition, sleep/fatigue)	HRmax-HRmin during DBT and NMSS score **r = −0.652**.	N	N
(Maetzler et al., 2015)	PD	Y	CS	45	N	65.6	44	MMSE 28.4	ECG	Resting supine during deep breathing cycles	Short term; 2 min	RMSSD, LF, HF, LF/HF, expiration/inspiration ratio (E/I ratio); pNN50	BDI	NR. “No significant correlations” between autonomic parameters and BDI.	N	N
*Studies not included in meta-analysis*																
(Mishima et al., 2005)	VD	Y	Pre-post	13	N	76.9	85	MMSE 17.4	ECG	NR	Long-term; “throughout the trial session” which lasted 6 days, and values averaged for each night.	LF, HF, LF/HF	Sleep parameters including sleep latency (time from bedtime to sleep-onset)	NR. Significant increases in HF and LF were accompanied by reductions in sleep latency associated with 2 days of passive body heating compared to baseline.	N	N
(Ino-Oka et al., 2005)	AD, VD	N	Observational	32	Severe congestive HF or IHD over NYHA class 2 and frequent arrhythmias	81.6	100	MMSE 14.5	ECG	Ambulatory	Long-term; 9 am-3 pm	RR interval, HF, LF/HF at 20 sec analysis intervals from 5 min prior to intentional behavior	Expectation control (increase in sympathetic tone at rest in preparation for subsequent behavior)	NR. RR and HF measures increased and LF/HF decreased prior to intentional behaviour.	N	N
(Lukhanina et al., 2008)	PD	N	CS	35	N	61.1	63	MMSE 26.9	ECG	p300 recording during acoustic vigilance task, in morning before 1 pm	Short-term; 5 min during rest and task	SDNN, RMSSD, (AMo, IT, IAB indicated predominance of sympathetic influences)	P300 EEG latency and Luriya’s short term memory test	Luriya’s test with Amo *avg r = −0.37*, IT *avg r = −0.39,* IAB *avg r = −0.41* (all p < 0.05). P300 latency with AMo r = 0.52, IT r = 0.36, IAB r = 0.37 (all p < 0.05).	N	N
(Niwa et al., 2011)	PD, HC	Y	CS	57	Cholinergic and norepinephrine agents, sleeping pills and antipsychotics. DM, MDD, other neurological conditions, abnormal MRI.	69.1	41.3	MMSE 27	ECG	Ambulatory	Long-term; 24hrs	LF, HF, TP, VLF; mean 5 min values calculated.	Rest activities as measured by actigraphy.	NR. Stated no correlation between HRV and rest activity, apart from HF and sleep episodes in bed (“data not shown”).	N	N
(Collins et al., 2012)	aMCI and non-aMCI, HC	Y	CS	133	MDD, bipolar affective disorder, schizophrenia, other neurological disorders, severe IHD and valvular heart disease, unstable tachycardia, history of drug or alcohol abuse and/or dependence in the previous 12 months.	72.6	47.5	MMSE 26.8	ECG	Resting supine between 9 am and 1 pm, abstained from caffeine, nicotine, vigorous exercise for > 12hrs	Short-term; 5 min (of 10 min recording)	Parasympathetic tests: HRmax-HRmin during deep breathing, HR response (longest RR interval at 30th beat:shortest RR interval at 15th beat) to orthostasis, valsalva ratio (RR interval expiration/RR ratio inspiration).	MMSE, CAMCOG-R, executive function, RT, working memory	NR. Participants were divided into 3 groups according to the number of abnormal parasympathetic tests (0,1, or >=2) and univariate analyses showed differences between groups in MMSE, CAMCOG-R, memory, exec function, RT, working memory. Multivariate analysis controlling for age, gender, medications, cardiovascular comorbidities showed differences between groups in MMSE, CAMCOG-R and working memory.	N	Y
(Yang et al., 2015)	AD, VD	Y	Controlled intervention	56, 73, 57	N	85.3, 83.7, 81.6	18, 34, 25	NR	ECG	After lunch in own rooms	NR	LF, HF, LF/HF	CMAI (agitation) scores	NR. One-way ANOVAs found that agitation reduced and parasympathetic activity increased over time in 2 treatment groups.	N	N
(Raglio et al., 2010)	AD, VD, mixed	Y	Controlled intervention	10, 10	Beta-blockers, atrial fibrillation	87, 84	70, 80	MMSE 13, 17	ECG	During wakefulness and sleep before and after a 15 week music therapy (MT) course or standard treatment	Long-term; 24hrs	SDANN, pNN50, SDRR, CV	NPI	NR. Stated 3 of the 5 patients who showed an improvement of pNN50 after MT also had a positive effect on NPI depression. After treatment, mean pNN50 increased slightly, but not significantly; pNN50 values improved in 50% patients of the MT group, but in none of the control group. The NPI depression sub-score significantly decreased after treatment vs controls	N	N
(Marshall et al., 2018)	FTD (bvFTD, nfPPA, rtvFTD, svPPA), HC	Y	Observational	51	Cardiac rate-limiting medications, cardiac arrhythmias.	67.6	41.1	MMSE 26	ECG	During emotional faces task	Short term; 20 min	Cardiac reactivity to viewing facial emotion was derived for each emotion as the percentage change in RR interval for three heart beats before and after the onset of each facial expression; averaged across all five emotions to provide a measure of overall reactivity.	Emotional reactivity across the FTD spectrum	NR. Increase in RR interval (cardiac deceleration) across the whole sample was found in response to viewing every emotion. ANOVA of cardiac reactivity incorporating all emotions showed a main effect of the participant group but not emotion type. Post hoc tests showed attenuated HR responses relative to healthy controls in the bvFTD group and nfvPPA group but not the rtvFTD group or svPPA group.	Y	Y - group membership, age
(Han et al., 2019)	“Dementia”, HC	N	Case-control	50, 34	History of psychiatric disease, substance abuse or dependence problems, neurological or medical diseases.	71.5, 71.1	12, 14.7	K-MMSE 12.8, 24.7	Bluetooth HR sensor wrapped around the patient’s chest	During video watching	Short term; 4 min	HF	HF changes in relation to funny, fearful, neutral emotions	NR. HF-HRV changes from baseline in response to funny stimulation (F = 4.04, P = 0.04) as well as from neutral to fear stimulation (F = 5.94, P = 0.02) in patients with dementia was increased, compared to the responses in the control group	N	N

Abbreviations: CS = cross-sectional; HRV = heart rate variability; ANS = autonomic nervous system; AD=Alzheimer’s disease, MCI = mild cognitive impairment; aMCI = amnestic MCI; VD = vascular dementia; HD = Huntington’s disease; FTD=fronto-temporal dementia; bvFTD= behavioral variant FTD; rtvFTD = right temporal variant FTD; nfPPA = non-fluent primary progressive aphasia; svPPA = semantic variant primary progressive aphasia; MS = Multiple Sclerosis; UHDRS=Unified Huntington’s Disease Rating Scale; NMSS = Non-Motor Symptom Scale; BDI = Beck Depression Inventory; HADS-A = Hospital Anxiety and Depression Scale - Anxiety; CMAI = Cohen-Mansfield Agitation Inventory; NPI = Neuropsychiatric Inventory; NEO-PI-3 = Neuroticism-Extraversion-Openness Personality Inventory-3; HC = healthy controls; EMG = electromyography; ECG = electrocardiography; RRIV = R-R interval variation; AChEIs = acetylcholinesterase inhibitors; TCA=tricyclic antidepressant; SSNRI = selective serotonin noradrenaline reuptake inhibitor; HF= heart failure; HTN = hypertension; IHD = ischaemic heart disease; DM = diabetes mellitus; MDD = major depressive disorder; CVD = cerebrovascular disease; TBI = traumatic brain injury; BMI = body mass index; RMSSD = Root Mean Square of Successive RR interval differences; LF = low frequency band power measure; ULF = ultra-low-frequency band power measure; VLF = very-low-frequency band power measure; HF = high frequency band power measure; LF/HF = LF/HF power ratio; TP =Total power (total of ULF, VLF, LF, and HF bands for 24 h and the VLF, LF, and HF bands for short-term recordings); SDRR = standard deviation of RR intervals; SDARR = standard deviation of 5 min average RR values for each 5 min interval; pNN50 = percentage of successive RR intervals that differ by more than 50 ms; SDRR idx = average of SDARR; Amo = “amplitude of mode of RR intervals”; IT = “index of tension of regulatory systems by Baevskii”; IAB = “index of autonomic balance by Baevskii”; IBI = interbeat interval; CV = coefficient of variation (SDRR/meanRR or SDNN/meanNN); RSA = respiratory sinus arrhythmia; DBT = deep breathing test; MMSE= Mini-Mental State Examination; CAMCOG(-R) = Cambridge Cognition Examination (- Revised); COWAT = Controlled Oral Word Association Test; TMT-B = Trail-Making Test B; IGT = Iowa Gambling Task; QUIP-RS = Questionnaire for Impulsive-compulsive disorders in Parkinson’s disease Rating Scale; CDR-SB= Clinical Dementia Rating Scale - Sum of Boxes; RT = reaction time; ACC= anterior cingulate cortex; OFC = orbitofrontal cortex; FI = frontoinsula; Y= yes; N = no; NR = not reported.

The most common neurodegenerative condition included in studies was amnestic MCI (in 9 studies), followed by AD (7 studies), PD (6 studies), vascular dementia (5 studies), FTD (2 studies), LBD (1 study), HD (1 study), multiple sclerosis (1 study). Non-amnestic MCI, including MCI-DLB, was additionally included in 3 studies. One study recruited participants with dementia but did not provide further diagnostic information. Most (20 of 27, 74%) studies used published diagnostic criteria to determine participant eligibility.

In the 20 studies that analyzed cross-sectional correlations between HRV indices and cognition/behavior, the average study size was N = 51 (SD 39) participants with a mean age of 69.2 (SD 10.0) years of whom 56.8% were female (one study did not report age or sex data). The mean MMSE (including values imputed from MoCA and CDR-SB scales) score was available from 15 of the 19 studies, which on average was 24.1 (SD 6.3). Two studies reporting cross-sectional associations between HRV and cognition/behavior included healthy controls in the sample.

For the seven studies that did not analyze cross-sectional associations between HRV and cognition/behavior measures, some reported between-group differences in task-related HRV change (Han et al., 2019, Marshall et al., 2018) or autonomic system dysfunction-related cognitive performance (Collins et al., 2012), and others reported HRV changes associated with a task or intervention (Ino-Oka et al., 2005), which were reported to occur alongside changes in cognition/behavior (Mishima et al., 2005, Raglio et al., 2010, Yang et al., 2015).

Most (19 of 27, 70%) studies described the exclusion of medications and comorbidities that could potentially impact the interpretation of HRV indices. Examples of excluded medications included antidementia drugs (acetylcholinesterase inhibitors and memantine), anticholinergic and noradrenergic agents, antidepressants, sleeping pills, antipsychotics, thyroxine and antihypertensives. Commonly excluded comorbidities were cardiovascular diseases (e.g. stroke, ischaemic heart disease, congestive heart failure, history of myocardial infarction, arrhythmia, history of cardiac surgery or implantation of a cardiac pacemaker, uncontrollable hypertension), diabetes, psychiatric illness e.g. major depression, prior drug dependence or abuse, and thyroid abnormalities. Only one study intentionally recruited participants with psychiatric comorbidity (high agitation scores) (Yang et al., 2015). No studies explicitly reported adjusting HRV values or analyses for baseline heart rate, which may have influenced the findings.

### 3.2 Measurement and outcomes

#### 3.2.1 HRV measurement

An electrocardiogram (ECG) was most commonly used to obtain heart rate measurements (22 of 27 studies, 81%), but some also employed a pulse oximeter (Guo et al., 2016, Sander et al., 2019), a Bluetooth heart rate sensor on the chest (Han et al., 2019), a wrist-worn monitor (Quinci and Astell, 2021) or electromyography (EMG) (Sharma et al., 1999). Eight studies specified a time range during which HRV was measured, which was typically in the morning (6 studies). Five studies also provided instructions for the participants to abstain from caffeine or nicotine, alcohol and/or vigorous exercise prior to the recording.

Around a quarter (7 of 27, 26%) of the studies measured resting HRV, i.e. during spontaneous breathing while supine or sitting and not during a cognitive task, and one study described measuring resting HRV but did not specify the posture of participants (Lin et al., 2017b). Of these 7 studies, 6 reported the duration of measurement, which ranged from 30 s to 16 min, to be on average approximately 7 min (mode was 5 min).

A similar proportion (6 of 27, 22%) of studies measured HRV change/reactivity to a cognitive task/stress, which was in the form of computerized tasks designed to test executive function (Stroop and Dual 1-back tasks) or processing speed and attention, or in response to viewing emotional faces. Three of these studies (Lin et al., 2017a, Lin et al., 2016, McDermott et al., 2019) expressed HRV change/reactivity using a quadratic model, with the quadratic term equating to rate of change. Alternative methods to express HRV change included the difference between HRV during task minus HRV during rest (Lin et al., 2017b), the change in HRV during task (after a cognitive intervention) whilst controlling for HRV during rest (Lin et al., 2020), or the change in HRV between the three heart beats before and after viewing emotional faces (Marshall et al., 2018).

Five studies measured ambulatory heart rate over several hours, most commonly 24 h (3 studies). From these longer-term recordings, two studies analyzed the mean HRV indices over 5 min and one analyzed HRV over 20 sond intervals in the 5 min prior to ‘intentional behavior’. Only three studies measured HRV during a cognitive task, which included an acoustic vigilance task (Lukhanina et al., 2008, Sander et al., 2019) and emotional video watching (Han et al., 2019). HRV was also sometimes reported to be measured during deep breathing cycles (5 studies), in relation to the valsalva manoeuvre (3 studies) and/or orthostasis, i.e. the heart rate response to standing from supine positions (2 studies).

#### 3.2.2 HRV indices

Although most (23 of 27, 85%) studies measured widely-used, HRV time (including RMSSD, pNN50, RSA, HRmax-HRmin) and frequency-domain (including HF, LF, LF/HF) indices, as described in an earlier review (Shaffer and Ginsberg, 2017), some studies obtained other measurements of HRV included the Expiration/Inspiration (E/I) ratio (longest R-R interval during expiration divided by shortest R-R interval during inspiration) (Del Pino et al., 2020, Maetzler et al., 2015), the Valsalva ratio (R-R interval during the procedure i.e. expiration divided by R-R interval during inspiration shortly after the procedure) (Collins et al., 2012, Del Pino et al., 2020), the heart rate response to orthostasis (longest R-R interval around the 30th beat divided by shortest RR interval around 15th beat) (Collins et al., 2012), and the R-R Interval Variation (RRIV) (difference between shortest and longest R-R interval divided by the mean of the shortest and longest intervals, expressed as a percentage) (Sharma et al., 1999). In addition, two studies used eponymous HRV indices from earlier published studies (e.g. ‘Index of autonomic balance by Baevskii’ (Lukhanina et al., 2008) and the ‘Toichi cardiac sympathetic and vagal indices’ (Guo et al., 2016)), and one study measured percentage change in R-R interval for the three beats before and after viewing emotional faces (Marshall et al., 2018).

The indices included in studies predominantly measured parasympathetic influences on HRV, as defined above, and only one of the 19 studies that reported any correlation between HRV and cognition/behavior solely described non-standard HRV indices that were interpreted to indicate sympathetic influences on autonomic function, but the calculation of these indices were not specified (Lukhanina et al., 2008).

#### 3.2.3 Cognitive/behavior measures

Over half (17 of 27, 63%) of the studies investigated the relationship between HRV and cognition, including executive functions, global cognition, memory, emotional reactivity, processing speed and attention. Fewer than half (12 of 27, 44%) of the studies investigated the relationship between HRV and behavior, including neuropsychiatric symptoms such as agitation (Yang et al., 2015), depression (Maetzler et al., 2015, Raglio et al., 2010), anxiety (Quinci and Astell, 2021), apathy and disinhibition (Guo et al., 2016), and UHDRS total behavior score (Sharma et al., 1999). Some studies assessed rest activities (Niwa et al., 2011) and sleep latency (Mishima et al., 2005), and self-reported chronic stress (McDermott et al., 2019) and cognitive fatigue (Lin et al., 2016, Sander et al., 2019) in relation to HRV. In one study, behavior and cognition for participants with PD were measured on a combined Non-Motor Symptom Scale (NMSS) (Bidikar et al., 2014).

#### 3.2.4 Neuroimaging findings

Of nine studies that reported additional neuroimaging findings associated with HRV, five described changes in functional connectivity between regions within the salience network (Guo et al., 2016, Lin et al., 2020), hippocampal network (McDermott et al., 2019), basal ganglia and central executive networks (Lin et al., 2016), and striatum-prefrontal network (Lin et al., 2017a). All of these reported that greater change or rate of change in HRV (quadratic term) was indicative of better adaptation capacity to stress and associated with stronger regional functional connectivity, apart from one study that described a higher HRV quadratic term as indexing worse acute stress adaptation, and found a negative correlation between hippocampal functional connectivity and HRV rate of change (McDermott et al., 2019).

Four studies reported on any structural brain differences in relation to HRV. Three studies in aMCI found no significant relationship between HRV and hippocampal (McDermott et al., 2019, Nicolini et al., 2020) or insula (Nicolini et al., 2020) volumes, or a measure of AD-related cortical atrophy in temporal and entorhinal cortices and fusiform gyrus (Lin et al., 2017b), although HRV reactivity was related to posterior insula, anterior cingulate and orbitofrontal cortical atrophy in several FTD subtypes (Marshall et al., 2018). One study found no association between HRV and nigrostriatal dopamine depletion in MCI-DLB participants using positron emission tomography (PET) imaging (Kim et al., 2018).

### 3.3 Main effect

**Fig. 2:**
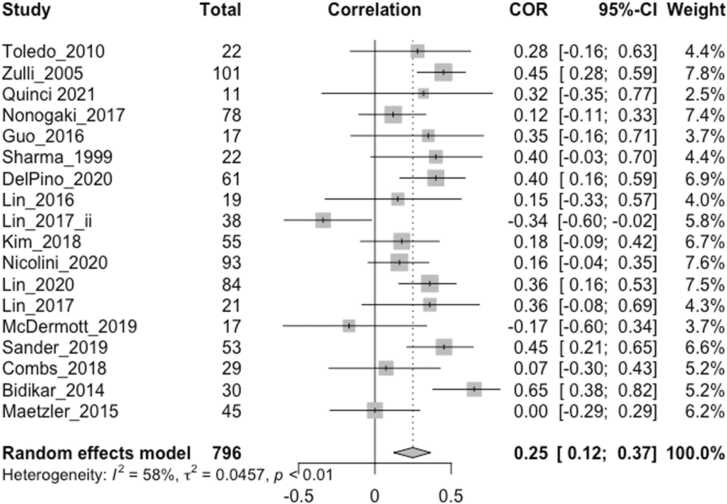
Forest plot showing Pearson correlation coefficients between vagally-mediated HRV and cognition/behavior measures reported by all included studies (N = 18). ‘Total’ denotes the sample size, COR and 95% CI denote the Pearson correlation coefficients and associated 95% confidence interval, and Weight is the adjusted random-effects weight using the Sidik-Jonkman estimator, for each study.

The effect sizes (Pearson’s r or beta coefficient within the range -/+0.5) of any cross-sectional correlation between vagally-mediated HRV indices and cognitive/behavioral measures from 18 studies, including the imputed effect size of one non-significant study without obtainable quantitative data (r = 0) (Maetzler et al., 2015), were included in the main meta-analysis (Fig. 2), with higher effect sizes indicating better cognitive/behavioral function. For the two studies that reported both resting HRV and task-related HRV reactivity (Lin et al., 2017b, Lin et al., 2016), only the values during rest were included in the main meta-analysis. For one study that stated a higher HRV quadratic term (greater rate of change) indexed worse acute stress adaptation (McDermott et al., 2019), which was in contrast to other similar studies where it indexed better adaptation capacity, the effect size was conservatively interpreted to be negative in this analysis for consistency.

The overall weighted effect size of the relation between vagally-mediated HRV and cognition/behavior measures was moderate and significant (r = 0.25, CI 0.12–0.37, p = 0.0001) (Fig. 2). The resting HRV effect sizes from two studies (Bidikar et al., 2014, Lin et al., 2017b) were detected to be outliers, and exclusion of these studies reduced the I^2^ heterogeneity value from 58.4% (p = 0.001) to 25.6%, which was no longer significant (p = 0.163), and slightly increased the pooled effect size to 0.26 (CI 0.16–0.36, p < 0.0001). Neither mean age (β = −0.0124, p = 0.0906) nor mean MMSE (β = −0.0115, p = 0.3814) predicted study effect size when included in separate meta-regression models, which also applied after excluding the two outliers.

### 3.4 Exploratory analyses

**Fig. 3:**
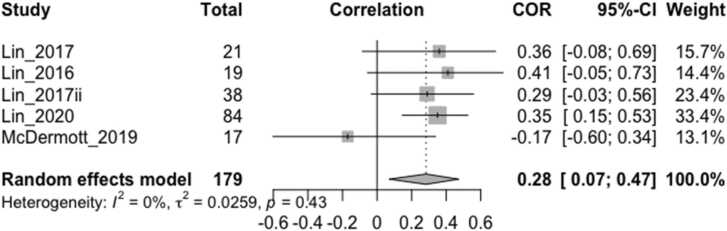
Forest plot showing Pearson correlation coefficients between task-related HRV reactivity/change and cognition/behavior measures. The participant population in all studies was amnestic MCI. Correlations were between HF-HRV rate of change (quadratic term) and processing speed, attention and working memory (Lin_2017 (Lin et al., 2017a)), reduced mental fatigability (Lin_2016 (Lin et al., 2016)) reduced perceived chronic stress severity (McDermott_2019 (McDermott et al., 2019)), between HF-HRV reactivity (HRV task minus HRV rest) and executive function (Lin_2017ii (Lin et al., 2017b), and between change in HR-HRV task (controlled for HRV rest) and processing speed and attention (Lin_2020 (Lin et al., 2020)). As McDermott_2019 stated that a higher HRV quadratic term (greater rate of change) indexed worse acute stress adaptation, which was in contrast to the other studies, it was conservatively interpreted to be the opposite (i.e. higher quadratic term indicating better adaptation) in this analysis for consistency. ‘Total’ denotes the sample size, COR and 95% CI denote the Pearson correlation coefficients and associated 95% confidence interval, and Weight is the adjusted random-effects weight using the Sidik-Jonkman estimator, for each study.

**Fig. 4:**
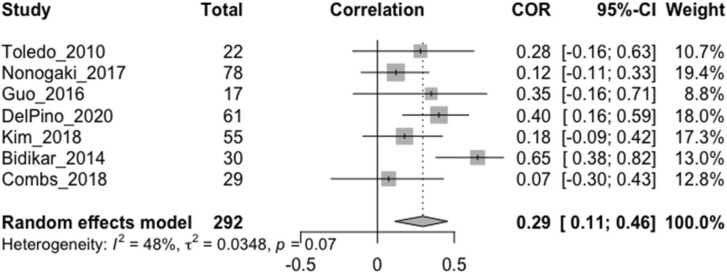
Forest plot showing Pearson correlation coefficients between resting vagally-mediated HRV and cognition/behavior measures. Only studies that reported measuring HRV during spontaneous breathing in a sitting or supine position were included. All studies employed ECG apart from Guo et al. (2016) which used pulse oximetry. ‘Total’ denotes the sample size, COR and 95% CI denote the Pearson correlation coefficients and associated 95% confidence interval, and Weight is the adjusted random-effects weight using the Sidik-Jonkman estimator, for each study.

The overall effect size estimate for the relationship between vagally-mediated HRV reactivity/change and cognition/behavior (Fig. 3), which was pooled from studies of aMCI participants, was comparable (r = 0.28, CI 0.07–0.47, p = 0.0099, I^2^ =0%) to vagally-mediated HRV during rest (r = 0.29, CI 0.11–0.46, p = 0.0017, I^2^ =48.2%) (Fig. 4). Exclusion of one resting HRV study that employed pulse oximetry instead of ECG (Guo et al., 2016) did not change the effect size.

**Fig. 5:**
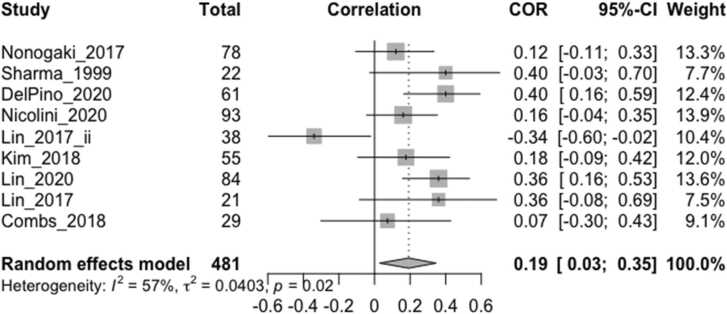
Forest plot showing Pearson correlation coefficients between vagally-mediated HRV and executive function measures. ‘Total’ denotes the sample size, COR and 95% CI denote the Pearson correlation coefficients and associated 95% confidence interval, and Weight is the adjusted random-effects weight using the Sidik-Jonkman estimator, for each study.

The overall effect size estimate for the relationship between vagally-mediated HRV and executive function (Fig. 5) was r = 0.19 (CI 0.03–0.35), which increased to r = 0.25 (CI 0.13–0.36) and study heterogeneity was eliminated, after the exclusion of one outlier (Lin et al., 2017b).

### 3.5 Study quality

Of the 20 studies that analyzed cross-sectional associations between HRV indices and cognition/behavior, most (13 studies) were judged to have some risk of bias, four had low risk and three had high risk of bias (Table 2). Almost all studies were judged to have clearly defined the research question, study population, and used valid HRV indices. Most studies did not report the time period over which the participants were recruited and it was often not possible to determine the participation or dropout rate from the reported data. The question on sample size and power calculation (domain 5) was only applied to seven studies that stated an a priori hypothesis or a prediction, but was only provided in one study; otherwise studies were presumed to be exploratory in design. The question on blinding (domain 12) was only applied to case-control or intervention studies. In line with the NIH Quality Assessment guide, domains 6 and 7 relating to exposure (cognition/behavior) and outcome (HRV) were not applicable to cross-sectional studies.

**Table 2:** Quality assessment using the NIH Quality Assessment Tool for cross-sectional and observational cohort studies.

Study	Quality assessment question														Quality rating	Risk of bias
	1	2	3	4	5	6	7	8	9	10	11	12	13	14		
(Sharma et al., 1999)	Y	Y	CD	NR	NA	NA	NA	Y	Y	N	Y	NA	CD	N	5 / 10	Some
(Zulli et al., 2005)	Y	Y	CD	NR	NA	NA	NA	Y	Y	N	Y	NA	CD	N	5 / 10	Some
(Lukhanina et al., 2008)	Y	Y	CD	NR	NA	NA	NA	Y	Y	N	N	NA	CD	N	4 / 10	High
(de Vilhena Toledo and Junqueira, 2010)	Y	Y	CD	NR	NA	NA	NA	Y	Y	N	Y	NA	CD	N	5 /10	Some
(Niwa et al., 2011)	Y	Y	Y	Y	NA	NA	NA	Y	Y	N	Y	NA	Y	N	8 /10	Low
(Bidikar et al., 2014)	Y	Y	CD	NR	NA	NA	NA	Y	Y	N	N	NA	Y	N	5 / 10	Some
(Maetzler et al., 2015)	Y	Y	CD	NR	NA	NA	NA	Y	Y	N	Y	NA	CD	N	5 / 10	Some
(Lin et al., 2016)	Y	N	CD	NR	N	NA	NA	Y	Y	Y	Y	NA	CD	Y	6 / 11	Some
(Guo et al., 2016)	Y	Y	CD	NR	N	NA	NA	Y	Y	N	Y	NA	Y	Y	7 /11	Some
(Nonogaki et al., 2017)	Y	Y	CD	NR	NA	NA	NA	Y	Y	N	Y	NA	CD	Y	6 / 10	Some
(Lin et al., 2017a)	Y	Y	CD	NR	N	NA	NA	Y	Y	N	Y	NA	Y	N	6 / 11	Some
(Lin et al., 2017b)	Y	Y	CD	NR	N	NA	NA	Y	Y	N	Y	NA	CD	Y	6 / 11	Some
(Kim et al., 2018)	Y	Y	Y	Y	NA	NA	NA	Y	Y	N	Y	NA	NA	Y	8 / 9	Low
(Combs et al., 2018)	Y	Y	CD	NR	NA	NA	NA	Y	Y	N	Y	NA	CD	N	5 / 10	Some
(McDermott et al., 2019)	Y	Y	CD	NR	N	NA	NA	Y	Y	N	Y	N	CD	N	5 / 12	High
(Sander et al., 2019)	Y	Y	CD	NR	N	NA	NA	Y	Y	N	Y	NA	Y	N	6 / 11	Some
(Nicolini et al., 2020)	Y	Y	Y	Y	NA	NA	NA	Y	Y	N	Y	NA	Y	Y	9 / 10	Low
(Del Pino et al., 2020)	Y	Y	CD	NR	NA	NA	NA	Y	Y	N	Y	NA	CD	Y	6 / 10	Some
(Lin et al., 2020)	Y	Y	CD	NR	Y	Y	Y	Y	Y	Y	Y	Y	CD	Y	11 / 14	Low
(Quinci, M.A., Astell, A.J., 2021)	Y	N	CD	NR	NA	NA	NA	Y	Y	N	Y	NA	CD	N	4 / 10	High

Abbreviations: Y = Yes, N = No, NA= not applicable, NR = not reported, CD = cannot determine. Questions were:

1) Was the research question or objective in this paper clearly stated?

2) Was the study population clearly specified and defined?

3) Was the participation rate of eligible persons at least 50%?

4) Were all the subjects selected or recruited from the same or similar populations (including the same time period)? Were inclusion and exclusion criteria for being in the study prespecified and applied uniformly to all participants?

5) Was a sample size justification, power description, or variance and effect estimates provided?

6) For the analyses in this paper, were the exposure(s) of interest measured prior to the outcome(s) being measured?

7) Was the timeframe sufficient so that one could reasonably expect to see an association between exposure and outcome if it existed?

8) For exposures that can vary in amount or level, did the study examine different levels of the exposure as related to the outcome (e.g., categories of exposure, or exposure measured as continuous variable)?

9) Were the exposure measures (independent variables) clearly defined, valid, reliable, and implemented consistently across all study participants?

10) Was the exposure(s) assessed more than once over time?

11) Were the outcome measures (dependent variables) clearly defined, valid, reliable, and implemented consistently across all study participants?

12) Were the outcome assessors blinded to the exposure status of participants?

13) Was loss to follow-up after baseline 20% or less?

14) Were key potential confounding variables measured and adjusted statistically for their impact on the relationship between exposure(s) and outcome(s)?

### 3.6 Small-study effects and publication bias

**Fig. 6:**
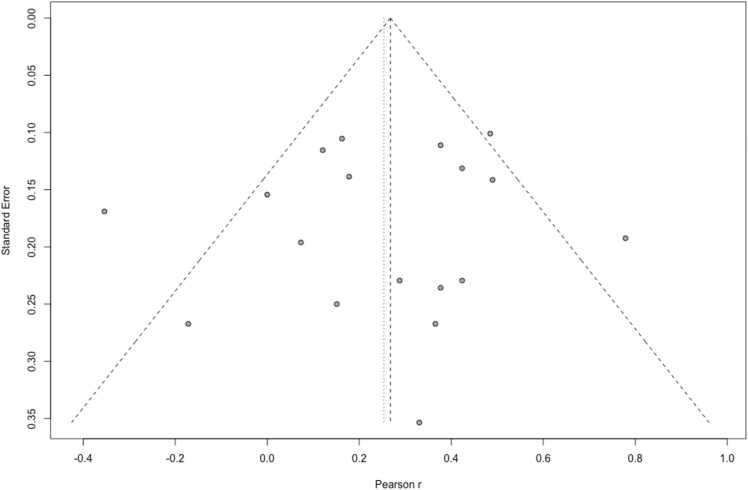
Funnel plot showing low risk of small sample bias across studies included in the meta-analysis. Plot asymmetry was not detected using Egger’s test (p = 0.627).

For the 18 studies included in the meta-analysis, a funnel plot (Fig. 6) showed a low degree of asymmetry, indicating low risk of small sample bias (i.e. small studies were not more likely to report significant and larger effects versus larger studies, a phenomenon usually attributed to publication/reporting bias), which was confirmed using Egger’s test for funnel plot asymmetry (p = 0.627).

Nine conference abstracts and one dissertation, which were not associated with published articles, reported on the association between HRV and cognition/behavior. Of these, five provided correlation coefficients between vagally-mediated HRV indices and measures of cognition/behavior (including an imputed value of r = 0 for two studies that reported no correlation), which were MMSE (2 studies in AD; no correlation), sleep efficiency (in MCI), and executive function (in amyotrophic lateral sclerosis/primary lateral sclerosis), and the weighted overall effect size was 0.14 (CI −0.12 to 0.38), which was not significant (p = 0.0839). The other five conference abstracts, for which correlation coefficients could not be extracted or imputed, reported significant associations between HRV indices and cognition and/or mood.

## 4 Discussion

Heart rate variability has been measured using a range of methods and in relation to a number of cognitive/behavioral outcomes in neurodegenerative conditions. The most commonly studied group was aMCI and short-term resting HRV or HRV change/reactivity was usually measured. Across 18 studies, there was a moderate and positive overall effect between vagally-mediated HRV indices and cognitive/behavioral outcomes (r = 0.25), which was slightly larger for HRV during rest (r = 0.30) compared to HRV change/reactivity (r = 0.28), and smaller for executive function (r = 0.19).

The associated neuroimaging findings, which were of stronger functional connectivity between central autonomic network regions and other functional networks in relation to higher HRV, supported a hierarchical model of vagal control involving the integration of different neural systems (Smith et al., 2017) and were consistent with neuroimaging findings in other populations (Mulcahy et al., 2019). The limited findings from structural MRI studies were mixed and may be condition-specific, as insula atrophy was found in FTD but not aMCI groups. Further research is needed to explore the interaction of the central autonomic network with other brain regions in relation to neuropsychiatric symptoms.

The effect sizes we found were larger than a previously reported pooled effect size of r = 0.09 between HRV and self-regulation measures across studies of mainly younger (<55 years) healthy adults (Holzman and Bridgett, 2017). This may be related to greater variability among the observations in older neurodegenerative patient groups. There were also differences in study characteristics, methodology and quality, and potential publication bias, which may have influenced outcomes.

### 4.1 Age, cognitive impairment, and other moderators

We found that the cross-sectional relationship between HRV indices and cognition/behavior across studies was not significantly influenced by mean age or MMSE. This is in contrast to an earlier study that found a stronger relationship between HRV and self-regulation measures with age (Holzman and Bridgett, 2017), although conclusions on older adults were limited as only five of 123 studies were in participants with a mean age of 55 years or older). It has been previously reported that vagally-mediated HRV declines with advancing age until at least the sixth decade of life, and may subsequently stabilize (De Meersman and Stein, 2007, Voss et al., 2012). On average, study participants were aged around 68 years and had MCI or mild dementia, so it is possible that we lacked sufficient power to detect any influence of older age or worse cognitive status on the reported effect sizes. Future studies involving participants with more severely impaired cognition and function, who may also have greater difficulty following task instructions and be more suited to resting or ambulatory HRV measurements, are needed to better understand the relationship between HRV and cognition/behavior in dementia.

A history of cardiovascular diseases, e.g. past myocardial infarction, cardiac failure and arrhythmias, diabetes, (Electrophysiology Task Force of the European Society of Cardiology the North American Society of Pacing, 1996), abnormal thyroxine levels and psychiatric illnesses such as depression (Kemp et al., 2010), can also influence HRV, but not all studies attempted to exclude or account for these conditions. Similarly, not all studies controlled for the use of common medications in neurodegenerative disorders, such as acetylcholinesterase inhibitors and levodopa, which could influence HRV by altering the balance of autonomic nervous system neurotransmitters, such as acetylcholine, which mediates the parasympathetic influence on heart rate, and noradrenaline, which mediates the sympathetic influence (Gordan et al., 2015).

### 4.2 HRV measurement factors

In order to standardize HRV measurements across studies, it has been recommended that short term recordings of 5 min or long-term recordings of 24 h should be used (Electrophysiology Task Force of the European Society of Cardiology the North American Society of Pacing, 1996, Laborde et al., 2017, Shaffer and Ginsberg, 2017). Although we found that the most common duration for resting HRV measurements was 5 min, this ranged from 30 s to 16 min, and was often longer for task-related HRV measures, which may have influenced the reported effect sizes. It has been recommended that at least one minute is required for HF-HRV measurement (Shaffer and Ginsberg, 2017). As short-term and long-term measures may not be completely interchangeable, this may have limited the precision of our overall pooled effect size (Fig. 2) which included one study that measured long-term HF-HRV over 24 h. The three exploratory meta-analyses (Fig. 3, Fig. 4, Fig. 5) included short-term HRV recordings only.

HRV can be influenced by environmental stimuli, as part of the adaptive response to stress, and by internal circadian rhythms, so it has been recommended to describe the testing environment (Electrophysiology Task Force of the European Society of Cardiology the North American Society of Pacing, 1996). However, only a minority of included studies did this and/or specified the time of day during which HRV was measured, which likely contributed to heterogeneity between studies. In addition, heart rate influences HRV and it has been proposed that the prognostic value and within-subject reproducibility of HRV can be improved if changes in heart rate are accounted for (Sacha, 2014), however no studies reported adjusting HRV values or analyses for baseline heart rate. Accurate HRV readings are also dependent on normal sinus rhythm and reasonable signal quality, so HRV data should undergo pre-processing and artifact correction (Laborde et al., 2017).

### 4.3 Outliers and publication bias

Findings from two studies in aMCI participants were inconsistent with the overall effect size that indicated higher HRV was associated with better measures of cognition/behavior (Lin et al., 2017b, McDermott et al., 2019). One of these reported a negative correlation between resting HF-HRV and executive function (r = −0.34) and cortical thickness in 38 aMCI participants (r = −0.37) (Lin et al., 2017b), although also reported positive correlations between HRV reactivity and these variables. The authors attributed this finding to an abnormally active parasympathetic nervous system, reflecting compensatory neural mechanisms, in response to AD-associated neurodegeneration in aMCI. However, this was not replicated in three other aMCI studies that reported positive effect sizes between resting HRV and cognitive/executive functions (Kim et al., 2018, Lin et al., 2016, Nicolini et al., 2020). Another study in aMCI participants found that higher HRV reactivity was associated with higher chronic stress severity scores, but also stated that a higher HF-HRV quadratic term indexed worse acute stress adaptation (McDermott et al., 2019), which was in contrast to other studies that stated the opposite, i.e a higher HF-HRV quadratic term indicated greater HRV reactivity and better stress adaptation. Further research, including longitudinal studies, in aMCI are needed to explore the extent to which any early neural compensatory mechanisms influence the relationship between HRV and cognition/behavior.

One study, detected to be an outlier in the meta-analysis, found a large positive correlation between HRmax-HRmin during deep breathing and lower Non-Motor Symptoms Scale (NMSS) score for 30 PD participants (r = 0.652) (Bidikar et al., 2014). This may have been related to the observation that smaller studies systematically report larger effects than larger studies, for example, due to publication bias, but small-study effects were not generally evident in our meta-analysis (Sterne and Harbord, 2004), based on the funnel plot analysis.

It was not possible to exclude the potential impact of publication bias on our findings. The inclusion of correlation coefficients from four conference abstracts and one dissertation in a post-hoc meta-analysis resulted in a small and non-significant pooled effect size, although other conference abstracts that reported significant findings did not provide quantitative data.

### 4.4 Other limitations

In order to examine the size and direction of any overall correlation between HRV and cognition/behavior in neurodegenerative disorders, we included several vagally-mediated HRV indices, obtained over a range of durations, in relation to a number of cognitive and behavioral measures from studies of different neurodegenerative disorders in our main meta-analysis (Fig. 2). Thus, these findings may lack precision with respect to specific HRV indices or cognition/behavior measures, although we conducted separate analyses to assess the specific effect sizes in relation to resting HRV (Fig. 4), HRV reactivity (Fig. 3) and executive function (Fig. 5). On the other hand, it is possible that our study could have been broader in its inclusion of potential vagally-mediated functions, as we did not include motor, autonomic or sensory symptoms, which may form part of a larger hierarchy of vagal control (Smith et al., 2017) and provided a more complete understanding of vagally-mediated HRV.

We lacked sufficient power to perform subgroup analyses within the neurodegenerative disorders, which would be important to study further as specific neurodegenerative disorders, e.g. LBD and MCI, may be associated with lower parasympathetic activity compared to others, such as AD (Cheng et al., 2020). Although we excluded studies of older adults with subjective memory complaints without a diagnosis of MCI, some individuals with MCI may not have had an underlying neurodegenerative condition, for example, their memory symptoms may have been due to a functional cognitive disorder (Ball et al., 2020), limiting the applicability of our findings to neurodegenerative disorders.

Heart rate variability is only one of several putative measures of sympathovagal function and this review is unlikely to provide a complete assessment of sympathovagal function in relation to cognition/behavior in neurodegenerative disorders. We aimed to systematically review the characteristics and methods of studies, but we did not assess the HRV data processing methods or equipment, for example, whether and how artifacts were reported to be removed or how spectral components of the HRV frequency-domain were analyzed. We also did not adjust findings for baseline heart rate, and heart rate data was not reported by all studies. These factors may have affected the accuracy and reliability of findings and contributed to heterogeneity across studies.

For the literature search, we used the search term “heart rate variability”, which is the conventionally accepted and widely-used term, and included in national guidelines (Electrophysiology Task Force of the European Society of Cardiology the North American Society of Pacing, 1996), so will likely have captured the vast majority of relevant studies. However, it is possible that some studies assessing inter-heart beat variation did not use this specific term and were missed. We excluded traumatic brain injury and vascular disease such as stroke, due to reported longer-term neural compensatory mechanisms post-insult in these conditions, and subsequently, greater difficulty interpreting and comparing the findings in relation to neurodegenerative disorders that involve decline in neural integrity over time.

Overall, we found evidence to support the concept that vagally-mediated indices of HRV are linked to cognitive and behavioral function in individuals with neurodegenerative conditions. It would be important for future studies to investigate this association in relation to neuropsychiatric symptoms and alongside neuroimaging methods, to elucidate the underlying neural networks and further explore the biomarker potential of HRV.

## Declaration of interests

None.
