# Supplementary material for: Heart rate variability in relation to cognition and behavior in neurodegenerative diseases: A systematic review and meta-analysis
Source: Ageing Res Rev. 2022 Jan;73:101539. doi: 10.1016/j.arr.2021.101539 (PMC8783051; doi:10.1016/j.arr.2021.101539)
Supplement: Supplementary file 1 — Supplementary material. [file mmc1.docx]

| Study | Neuroimaging measure(s) | Correlation statistic (neuroimaging) |
| --- | --- | --- |
| Lin 2016 | Basal ganglia network (BGN) and the central executive network (CEN). Inter-network connectivity between bilateral putamen from the BGN and bilateral middle frontal gyrus (MFG) from the CEN. | No sig. association between BGN and CEN networks and HRV indices r= 0.39 – 0.54 (p > 0.10). Left putamen-right MFG connectivity was related to vertex, r = −0.79, n = 7 (p = 0.018) and change, r = 0.79, n = 7 (p = .018) of HF-HRV model. |
| Guo 2016 | Resting fMRI correlation seed-based ROI (frontoinsula - FI) analyses with CVI or lnHRV as regressor, shown in R or L hemisphere for R handed subjects. | Reduced (vagally-mediated) HRV and BOLD signal coupling in bvFTD vs controls in the subgenual ACC and left FI. |
| Lin 2017 | fMRI resting state network analysis using bilateral striata as seeds. | Striatum-prefrontal network changes correlated with the quadratic term of HF-HRV responses (Left: r = 0.41, Right: r = 0.55). Sample size=11. |
| Lin 2017_ii | AD signature cortical thickness (ADSCT) defined by averaging cortical thickness of bilateral inferior and middle temporal lobes, entorhinal cortex and fusiform gyrus, a lower value indicating worse AD pathology | ADCST and resting HF-HRV r=-0.37; HF-HRV reactivity r=0.27; |
| Kim 2018 | 19 MCI-DLB subjects performed FP-CIT PET | No correlation between HRV and nigrostriatal dopamine depletion |
| Marshall 2018 | ROIs of central autonomic control network were ACC, insula, OFC | NR. Relative to controls, bvFTD reduced HR reactivity to emotion ass. with reduced gray matter in R dorsal ACC and left OFC, and in nfvPPA this was ass. with posterior insula atrophy. |
| McDermott 2019 | Resting state fMRI using R and L hippocampi as seeds. | Across the whole sample, hippocampal volumes did not correlate with HF-quadratic (L: r = −0.18; R: r = −0.16).  In the aMCI group, L hippocampus-R insula functional connectivity was correlated to the HF-quadratic, r = −0.68, and L hippocampus-L precentral gyrus connectivity was correlated to HF-quadratic, r = −0.55. No significant correlation in R hippocampal functional connectivity. No correlation with hippocampal volumes. |
| Nicolini 2020 | Insula and hippocampal atrophy and DWML burden scores | For aMCI, ΔLFn and ΔLF/HF exhibited a significant negative correlation with hippocampal (r=-0.331) insula (r=-0.326- -0.358) atrophy. HF r=0.027 (n.s.) hippocampus r=-0.071 insula (n.s.)  For naMCI, ΔLFn and ΔLF/HF exhibited a significant positive correlation with DWML burden (r=0.274-0.277). HF r=-0.168 (n.s.). |
| Lin 2020 | Functional connectivity (FC) in salience network (SN) | Across two MCI groups from baseline to post-test, greater FC strength in SN (but not other networks) over time was related to improvement in HF-HRV_task when controlling for HRV_rest. (B ​= ​0.03, corrected p ​< ​.001), These results did not change when controlling for age, sex, and a AD-related cortical thickness score (B ​= ​0.04, corrected p ​< ​.001). |

**Supplemental Table 1: Neuroimaging findings from included studies.** Abbreviations: HRV = heart rate variability; AD=Alzheimer’s disease, DLB = dementia with Lewy bodies; MCI = mild cognitive impairment; aMCI = amnestic MCI; bvFTD= behavioral variant FTD; nfPPA = non-fluent primary progressive aphasia; LF = low frequency band power measure; ULF = ultra-low-frequency band power measure; HF = high frequency band power measure; LF/HF = LF/HF power ratio; IBI = interbeat interval; CVI = Toichi cardiac vagal index; ACC= anterior cingulate cortex; OFC = orbitofrontal cortex; FI = frontoinsula; DWML = deep white matter lesions, ROI = region of interest.
